# Guidelines in dispute: what is at stake when “every meal” becomes the rule

**DOI:** 10.1590/S2237-96222026v35e20260184.en

**Published:** 2026-05-29

**Authors:** Pamela Gracielle da Fonseca, Maria Auxiliadora Parreiras Martins, Nathalia Sernizon Guimarães

**Affiliations:** 1 Universidade Federal de Minas Gerais Departamento de Nutrição Belo Horizonte MG Brazil Universidade Federal de Minas Gerais, Departamento de Nutrição, Belo Horizonte, MG, Brazil; 2 Universidade Federal de Minas Gerais Departamento de Produtos Farmacêuticos Belo Horizonte MG Brazil Universidade Federal de Minas Gerais, Departamento de Produtos Farmacêuticos, Belo Horizonte, MG, Brazil

National dietary guidelines are governance instruments with population-level effects. They guide health, food, and nutrition policies, influence food systems and consumption patterns, and consequently affect the environmental impacts of food production, consumption, and disposal. These effects are not limited to individual counseling, as guidelines inform health literacy actions, public procurement, and institutional standards for food provision, where recommendations are translated into routine decisions about meal planning, portioning, and provision [1].

In this context, recent communication framing associated with the Dietary Guidelines for Americans 2025–2030 has gained prominence, disseminated under the slogan “*Eat real food*” and accompanied by the message that every meal should prioritize high-quality protein, with elevated targets and emphasis on animal-source foods [2]. Although directed to the United States, this type of message tends to circulate as a technical and symbolic reference and to influence professional training, educational materials, and public communication. For Brazil, the critical point is not to import an external controversy, but to recognize that international normative frameworks may reorient social expectations regarding what counts as an “adequate meal” and, by extension, influence institutional choices and agenda disputes in the promotion of healthy eating.

From a public health perspective, the decisive issue lies in understanding how recommendations are translated into practice. In Brazil, this occurs mainly through collective food-provision environments, such as schools, health services, universities, and food and nutrition security equipment, where menus and public procurement promote large-scale changes. When “protein” is taken as the axis of adequacy, the likelihood of meal reconfiguration increases, with an expanded centrality of animal-source items, which has repercussions for sustainability and cultural equity and challenges the social acceptance of plant-based dietary patterns. At this menu-centered level, guidelines operate as an organizing force of the service, with cumulative effects on ingredient choices, portion sizes, and purchasing practices [1].

This debate should be situated within the architecture of multilateral commitments. The 2030 Agenda for Sustainable Development, adopted by the United Nations in 2015, presupposes coherence among policy instruments aimed at health, food security, consumption and production patterns, and climate action [3]. Evidence on its implementation indicates that achieving the targets depends on multilevel action and bottom-up trajectories, including consumption transitions associated with more sustainable diets [3]. Dietary guidelines, therefore, are not neutral; they may facilitate or hinder the convergence between health protection and promotion and the reduction of socioenvironmental pressures.

A normative framework centered on protein may produce undesirable social effects. Although both animal and plant sources are mentioned, the prescription to “prioritize protein in every meal” and the symbolic emphasis on animal-source items may function as markers of dietary normality and reinforce the perception that vegetarian and vegan diets are “restrictive” or “inadequate” [4]. There is evidence of bias and prejudice against vegetarians and, especially, vegans, including more negative evaluations by omnivores and experiences of stigma [4]. The literature describes “vegaphobia” as a pattern of ridicule and delegitimization of veganism in public discourse, with effects on the willingness to adopt or express plant-based dietary choices [5]. Given that dietary guidelines underpin health communication and food programs, recommendations should avoid formulations that stigmatize or exacerbate inequalities.

The effects of guidelines become particularly visible in food services. The reorientation of menus may affect waste, costs, and sustainability, especially in systems that serve large volumes of meals [1]. A systematic review and meta-analysis on food waste in food services identified a high magnitude of waste. They described the most effective strategies as those focused on clients, plate service rather than tray service, and actions such as food and nutrition education, portion adjustments, and measures to improve menu acceptance [6]. This finding is consistent with the idea that changes in service design are more scalable than individual appeals and that normative recommendations may reconfigure institutional routines with material consequences.

There are also specific implications for Indigenous peoples in Brazil, whose food systems depend on territory, seasonality, and biodiversity. National frameworks such as the Brazilian Policy for Territorial and Environmental Management of Indigenous Lands (*Política Nacional de Gestão Territorial e Ambiental de Terras Indígenas*, PNGATI) link territorial protection and the sustainable use of resources to physical and cultural reproduction and quality of life [7]. Within the Brazilian Unified Health System (*Sistema Único de Saúde*, SUS), the Indigenous Health Care Subsystem (*Subsistema de Atenção à Saúde Indígena*, SasiSUS) and the National Policy on Health Care of Indigenous Peoples (*Política Nacional de Atenção à Saúde dos Povos Indígenas*, PNASPI) reinforce the need for cultural adequacy and dialogue with traditional knowledge. This issue has gained prominence in the recent debate on updating the policy [8]. From the perspective of food and nutrition security, the National Survey on Food Sovereignty and Food and Nutrition Security of Indigenous Peoples (*Pesquisa Nacional sobre Soberania e Segurança Alimentar e Nutricional dos Povos Indígenas no Brasil*, PENSSAI) in Brazil highlights the importance of producing contextualized information to support public policies [9]. Recent syntheses describe the coexistence of hunger, anemia, food insecurity, and obesity among Indigenous peoples in the country and emphasize structural determinants and the limits of strictly biometric responses [10]. This debate is also grounded in international frameworks of collective rights, such as the United Nations Declaration on the Rights of Indigenous Peoples [11], and in the notion of Indigenous food sovereignty, which emphasizes self-determination over food systems, sources, and processes [12]. In this context, guidelines that reduce health to universal macronutrient targets may reinforce epistemic asymmetries and obscure the territorial and collective dimension of food.

At the environmental level, the accumulation of evidence reinforces the need for explicit planetary health criteria in recommendations. Studies have indicated that shifts in food demand may generate co-benefits for health and climate [13] and that maintaining consumption patterns incompatible with environmental limits strains the food system [14]. International analyses link diets to environmental sustainability [15] and suggest healthier, more sustainable dietary trajectories [16]. Reports from the Intergovernmental Panel on Climate Change highlight the relevance of changes in food demand as mitigation measures in the land-use sector, with environmental and health co-benefits [17,18]. Therefore, national guidelines that encourage greater reliance on foods with higher environmental impacts tend to exacerbate contradictions between health and climate objectives.

In Brazil, the Dietary Guidelines for the Brazilian Population (*Guia Alimentar para a População Brasileira*) is not merely a technical reference but a public policy framework with direct implications for the organization of food provision [19]. By structuring recommendations around foods, culinary practices, and the degree of processing, as defined by the NOVA classification, the guideline explicitly states that reducing the consumption and production of animal-source foods helps lower emissions, deforestation, and intensive resource use [19]. 

This framework becomes even more strategic in the face of the expansion of ultra-processed foods and their potential to displace traditional dietary patterns, with significant impacts on public health [20]. In systems in which menus and public procurement define what reaches the plate, messages that recenter dietary adequacy on “protein” and symbolically reinforce animal sources are not rhetorical details; they may reorganize institutional routines and produce lasting effects on equity and sustainability [1,6]. 

In this context, discussing sustainability around “protein” is inevitable, considering not only its nutritional value but also the environmental and social impact of its production chains. The issue goes beyond protein sufficiency and encompasses the origins and associated costs: animal proteins require intensive use of land, water, and feed, as well as significant emissions and logistical burdens. Plant alternatives, however, may involve monocultures, excessive processing, and long supply chains, which can compromise their sustainability. The central debate is not between animal- and plant-based. Still, the dietary pattern promoted includes diversity of sources, reduced waste, fresh or minimally processed foods, cultural adaptation, and transparency in supply chains. Protein sustainability, therefore, requires reconfiguring the food system as a whole [1,6].

For this reason, engaging in dialogue and constructing dietary guidelines is equivalent to debating the very design of food and nutrition policies in the broader context and within the SUS, linking them to the promotion of public health, coherence with climate commitments, and the protection of ways of life and collective rights. The core of the debate is not protein itself, but the governance model that becomes consolidated when recommendations cease to explicitly integrate population health—in terms of the universality and equity advocated by the SUS—environmental sustainability, and social justice.
