# Supplementary material for: Guidelines in dispute: what is at stake when “every meal” becomes the rule
Source: Epidemiol Serv Saude. 2026 May 29;35:e20260184. doi: 10.1590/S2237-96222026v35e20260184.en (PMC13686409; doi:10.1590/S2237-96222026v35e20260184.en)
Supplement: Translation

# Translation: Diretrizes em disputa: o que está em jogo quando “toda refeição” vira regra

**DOI:** 10.1590/S2237-96222026v35e20260184.pt

**Authors:** Pamela Gracielle da Fonseca, Maria Auxiliadora Parreiras Martins, Nathalia Sernizon Guimarães

**Affiliations:** 1 Universidade Federal de Minas Gerais Departamento de Nutrição Belo Horizonte MG Brasil Universidade Federal de Minas Gerais, Departamento de Nutrição, Belo Horizonte, MG, Brasil; 2 Universidade Federal de Minas Gerais Departamento de Produtos Farmacêuticos Belo Horizonte MG Brasil Universidade Federal de Minas Gerais, Departamento de Produtos Farmacêuticos, Belo Horizonte, MG, Brasil

Diretrizes alimentares nacionais são instrumentos de governança com efeitos populacionais. Elas orientam políticas de saúde, alimentação e nutrição, influenciam sistemas alimentares e padrões de consumo e, consequentemente, repercutem nos impactos ambientais associados à produção, ao consumo e ao descarte de alimentos. Esses efeitos não se limitam ao aconselhamento individual, pois diretrizes se concretizam em ações de letramento em saúde, compras públicas e padrões institucionais de oferta alimentar, nos quais recomendações se transformam em decisões rotineiras sobre planejamento, porcionamento e provisão de refeições [1].

Nesse cenário, ganha relevância o enquadramento comunicacional recente associado às *Dietary Guidelines for Americans 2025*-2030 (*Diretrizes Alimentares para os Americanos 2025*-2030) divulgado sob o slogan “*Eat real food*” (“Coma comida de verdade”) e acompanhado da mensagem de que toda refeição deve priorizar proteína de alta qualidade, com metas elevadas e destaque para fontes de origem animal [2]. Embora dirigido aos Estados Unidos, esse tipo de mensagem tende a circular como referência técnica e simbólica e a influenciar formação profissional, materiais educativos e comunicação pública. Para o Brasil, o ponto crítico não é importar uma controvérsia externa, mas reconhecer que marcos normativos internacionais podem reorientar expectativas sociais sobre o que conta como “refeição adequada” e, por extensão, influenciar escolhas institucionais e disputas de agenda na promoção da alimentação saudável.

Na perspectiva da saúde pública, a questão decisiva reside em compreender como as recomendações se convertem em prática. No Brasil, isso ocorre sobretudo por meio de ambientes de provisão alimentar coletiva, como escolas, serviços de saúde, universidades e equipamentos de segurança alimentar e nutricional, onde cardápios e compras públicas promovem mudanças em larga escala. Quando a “proteína” é tomada como eixo de adequação, cresce a probabilidade de reconfiguração das refeições, com centralidade ampliada de itens de origem animal, o que implica em repercussões para a sustentabilidade e a equidade cultural, além de tensionar a aceitação social de padrões alimentares baseados em plantas. Nesse nível, centrado no cardápio, as diretrizes operam como força organizadora do serviço, com efeitos cumulativos em escolhas de ingredientes, tamanhos de porções e práticas de compra [1].

Esse debate deve ser situado na arquitetura de compromissos multilaterais. A Agenda 2030 para o Desenvolvimento Sustentável, aprovada pela Organização das Nações Unidas em 2015, pressupõe coerência entre instrumentos de política voltados a saúde, segurança alimentar, padrões de consumo e produção e ação climática [3]. Evidências sobre sua implementação indicam que o alcance das metas depende de ação multinível e trajetórias construídas de baixo para cima, com inclusão de transições de consumo associadas a dietas mais sustentáveis [3]. As diretrizes alimentares, portanto, não são neutras, podem facilitar ou dificultar a convergência entre proteção e promoção da saúde e a redução de pressões socioambientais.

O enquadramento normativo centrado em proteína pode gerar efeitos sociais indesejáveis. Ainda que se mencionem as fontes animais e as vegetais, a prescrição de “priorizar proteína em toda refeição” e o destaque simbólico a itens de origem animal podem funcionar como marcadores de normalidade alimentar e reforço da percepção de que dietas vegetarianas e veganas são “restritivas” ou “inadequadas” [4]. Há evidências de viés e preconceito contra vegetarianos e, sobretudo, veganos, inclusive de avaliações mais negativas por onívoros e experiências de estigma [4]. A literatura descreve “vegaphobia” como padrão de ridicularização e deslegitimação do veganismo em discursos públicos, com efeitos sobre a disposição de adotar ou expressar escolhas alimentares baseadas em plantas [5]. Com a consideração de que diretrizes alimentares fundamentam comunicação em saúde e programas alimentares, as recomendações devem evitar formulações que favoreçam estigmatizações ou ampliem desigualdades.

Os efeitos das diretrizes tornam-se particularmente visíveis nos serviços de alimentação. A reorientação de cardápios pode impactar em desperdício, custos e sustentabilidade, sobretudo em sistemas que servem grandes volumes de refeições [1]. Uma revisão sistemática e meta-análise sobre desperdício em serviços de alimentação identificou elevada magnitude de desperdício e descreveu estratégias mais efetivas associadas ao foco nos clientes, ao serviço no prato em vez de em bandeja e a ações como educação alimentar, ajustes de porções e medidas de aceitação do cardápio [6]. Esse achado é coerente com as ideias de que mudanças no desenho do serviço são mais escaláveis do que apelos individuais e de que recomendações normativas podem reconfigurar rotinas institucionais com consequências materiais.

Há também implicações específicas para povos indígenas no Brasil, cujos sistemas alimentares dependem de território, sazonalidade e biodiversidade. Marcos nacionais como a Política Nacional de Gestão Territorial e Ambiental de Terras Indígenas vinculam proteção territorial e uso sustentável de recursos à reprodução física e cultural e à qualidade de vida [7]. No Sistema Único de Saúde (SUS), o Subsistema de Atenção à Saúde Indígena e a Política Nacional de Atenção à Saúde dos Povos Indígenas reforçam a necessidade de adequação cultural e de diálogo com saberes tradicionais, tema que ganha centralidade no debate recente de atualização da política [8]. Do ponto de vista da segurança alimentar e nutricional, a Pesquisa Nacional sobre Soberania e Segurança Alimentar e Nutricional dos Povos Indígenas no Brasil aponta a importância de produzir informações contextualizadas para subsidiar políticas públicas [9]. Sínteses recentes descrevem a coexistência de fome, anemia, insegurança alimentar e obesidade entre povos indígenas no país e enfatizam determinantes estruturais e limites de respostas estritamente biométricas [10]. Esse debate se ancora também em referenciais internacionais de direitos coletivos, como a Declaração das Nações Unidas sobre os Direitos dos Povos Indígenas [11], e na noção de soberania alimentar indígena, que enfatiza autodeterminação sobre sistemas alimentares, fontes e processos [12]. Nesse contexto, diretrizes que reduzem saúde a metas universais de macronutrientes podem reforçar assimetrias epistêmicas e obscurecer a dimensão territorial e coletiva da alimentação.

No plano ambiental, o acúmulo de evidências reforça a necessidade de critérios explícitos de saúde planetária nas recomendações. Estudos indicaram que mudanças na demanda alimentar podem gerar cobenefícios em saúde e clima [13] e que manter padrões de consumo incompatíveis com limites ambientais tensiona o sistema alimentar [14]. Análises internacionais relacionam dietas e sustentabilidade ambiental [15] e apontam possibilidades de trajetórias alimentares mais saudáveis e sustentáveis [16]. Relatórios do Painel Intergovernamental sobre Mudanças Climáticas destacam a relevância de mudanças na demanda alimentar como medidas de mitigação no setor de uso da terra, com cobenefícios ambientais e em saúde [17,18]. Assim, diretrizes nacionais que induzam mais dependência de alimentos de maior impacto ambiental tendem a ampliar contradições entre objetivos sanitários e climáticos.

No Brasil, o *Guia Alimentar para a População Brasileira* não é apenas um referencial técnico, mas um marco de política pública com implicações diretas para a organização da oferta alimentar [19]. Ao estruturar recomendações por alimentos, culinária e grau de processamento, com base na classificação NOVA, o guia explicita que reduzir consumo e produção de alimentos de origem animal contribui para reduzir emissões, desmatamento e uso intensivo de recursos [19]. 

Esse marco torna-se ainda mais estratégico diante do avanço dos ultraprocessados e do seu potencial de deslocar padrões alimentares tradicionais, com impactos relevantes para a saúde coletiva [20]. Em sistemas nos quais cardápios e compras públicas definem o que chega ao prato, mensagens que recentram a adequação alimentar em “proteína” e reforçam simbolicamente fontes animais não são detalhes retóricos; elas podem reorganizar rotinas institucionais e produzir efeitos duradouros sobre equidade e sustentabilidade [1,6]. 

Nesse contexto, discutir sustentabilidade em torno da “proteína” é inevitável, com a consideração não só seu valor nutricional, mas também do impacto ambiental e social das suas cadeias produtivas. A questão vai além da suficiência proteica, abrange origem e custos associados: proteínas animais demandam intensivo uso de terra, água, emissões, ração e logística. Alternativas vegetais, porém, podem envolver monoculturas, processamento excessivo e cadeias longas, o que compromete suas sustentabilidades. O debate central não é animal versus vegetal, mas o padrão alimentar promovido, com diversidade de fontes, menor desperdício, alimentos in natura ou minimamente processados, adaptação cultural e transparência nas cadeias. Sustentabilidade proteica exige, assim, reconfiguração do sistema alimentar como um todo [1,6].

Por isso, dialogar e construir diretrizes alimentares equivale a debater o próprio desenho das políticas de alimentação e de nutrição no contexto geral e do SUS, com articulação delas à promoção da saúde pública, à coerência com compromissos climáticos e à proteção de modos de vida e direitos coletivos. O centro do debate não é a proteína em si, mas o modelo de governança que se consolida quando recomendações deixam de compor, de forma explícita, a integração entre saúde populacional – em termos da universalidade e da equidade preconizadas pelo SUS –, sustentabilidade ambiental e justiça social.
